# *NTRK* Fusions in a Sarcomas Series: Pathology, Molecular and Clinical Aspects

**DOI:** 10.3389/pore.2022.1610423

**Published:** 2022-05-11

**Authors:** Vasiliki Siozopoulou, Elly Marcq, Koen De Winne, Koen Norga, Gertjan Schmitz, Valerie Duwel, Philippe Delvenne, Evelien Smits, Patrick Pauwels

**Affiliations:** ^1^ Department of Pathology, Antwerp University Hospital, Edegem, Belgium; ^2^ Center for Oncological Research (CORE), Integrated Personalized and Precision Oncology Network (IPPON), University of Antwerp, Wilrijk, Belgium; ^3^ Department of Pediatrics, Antwerp University Hospital, Edegem, Belgium; ^4^ Department of Orthopaedics, Hospital of Klina, Antwerp, Belgium; ^5^ Department of Pathology, Hospital of Klina, Antwerp, Belgium; ^6^ Department of Anatomopathology, CHU Sart Tilman, Liège, Belgium; ^7^ Center for Cell Therapy and Regenerative Medicine, Antwerp University Hospital, Edegem, Belgium

**Keywords:** sarcoma, prognosis, NTRK fusion, TRK immunohistochemistry, NTRK fusion partner, histology

## Abstract

Targeting molecular alterations has been proven to be an inflecting point in tumor treatment. Especially in recent years, inhibitors that target the tyrosine receptor kinase show excellent response rates and durable effects in all kind of tumors that harbor fusions of one of the three neurotrophic tyrosine receptor kinase genes (*NTRK1*, *NTRK2* and *NTRK3*). Today, the therapeutic options in most metastatic sarcomas are rather limited. Therefore, identifying which sarcoma types are more likely to harbor these targetable *NTRK* fusions is of paramount importance. At the moment, identification of these fusions is solely based on immunohistochemistry and confirmed by molecular techniques. However, a first attempt has been made to describe the histomorphology of *NTRK*-fusion positive sarcomas, in order to pinpoint which of these tumors are the best candidates for testing. In this study, we investigate the immunohistochemical expression of pan-TRK in 70 soft tissue and bone sarcomas. The pan-TRK positive cases were further investigated with molecular techniques for the presence of a *NTRK* fusion. Seven out of the 70 cases showed positivity for pan-TRK, whereas two of these seven cases presented an *NTRK3* fusion. Further analysis of the fused sarcomas revealed some unique histological, molecular and clinical findings. The goal of this study is to expand the histomorphological spectrum of the *NTRK*-fused sarcomas, to identify their fusion partners and to correlate these parameters with the clinical outcome of the disease. In addition, we evaluated the immunohistochemical expression pattern of the pan-TRK and its correlation with the involved *NTRK* gene.

## Introduction

Soft tissue and bone tumors are a very heterogenous group of neoplasms which makes prognosis difficult to assess. Moreover, given their rarity of occurrence, standardization of diagnostic criteria and precise treatment protocols is challenging. The majority of localized sarcomas are treated with excision mostly followed by adjuvant radiotherapy to control local recurrence [1, 2]. Few sarcoma types are chemo-sensitive, such as rhabdomyosarcoma, osteosarcoma and Ewing sarcoma [2, 3]. In the metastatic setting, the 5-year overall survival rates do not exceed 20%, highlighting that effective treatment of advanced disease remains a challenge. There is clear need for new therapeutic options in advanced sarcomas [4].

Some of the molecular alterations that are found in tumors are druggable and this has been a significant turning point in cancer treatment. One of the most important representatives are tyrosine kinase inhibitors (TKI) that are used for the treatment of diverse tumor types, such as imatinib for gastrointestinal stromal tumors (GIST) [5]. Unfortunately, today most of the oncogenic driver alterations remain undruggable.

Very recently, it has been proven that tumors with fusions of one of the three neurotrophic tyrosine receptor kinase genes (*NTRK1*, *NTRK2* and *NTRK3*) can be treated with tyrosine receptor kinase (TRK) -inhibitors. The first generation TRK-inhibitors, larotrectinib and entrectinib [6], show excellent response rates and durable effects in tumors that harbor fusion of one of the *NTRK* genes, regardless of tumor type or site of origin. The adverse events are well tolerated. Still, a major obstacle for those inhibitors is the development of resistance. Therefore, new generation TRK-inhibitors are developed, such as LOXO-195 and repotrectinib [7, 8].

Despite the promising results of TRK-inhibition, *NTRK* fusions are rare genetic events. Among sarcomas, infantile fibrosarcomas show fusions of the *NTRK3* gene in more than 90% of the cases [9]. The rates of *NTRK* fusions in the remaining sarcoma types are unfortunately very low. Moreover, no clear morphologic criteria are established for the recognition of the *NTRK*-fused sarcomas. A NTRK fusion has to be demonstrated by means of molecular analysis. Immunohistochemistry can be performed as an enrichment strategy to select tumors for subsequent molecular analysis In a previous publication, we suggested an algorithm for determining which sarcomas are more likely to carry this fusion [10].

In this study, we investigated the expression of the pan-TRK antibody in different types of soft tissue and bone tumors by immunohistochemistry and we correlated the expression of the positive cases with the presence of *NTRK* fusions. Furthermore, we summarized the clinicopathological characteristics, in an attempt to give more information about the identification of these tumors, in conjunction with the cases that are described in the literature.

## Materials and Methods

### Tissue Samples

Archival formalin-fixed paraffin embedded (FFPE) tissue samples from 70 patients with soft tissue and bone tumors were retrieved from the Department of Pathology at the Antwerp University Hospital. The tissue samples were collected from biopsy material as well as excision specimens. The material was not older than 5 years, to avoid loss of immunoreactivity and to obtain quality material for the additional molecular analysis. The biopsies were fixed in 4% formaldehyde for up to 12 h while the excision samples were fixed for up to 32 h.

Inclusion criteria where all locally aggressive and malignant soft tissue tumors, except infantile fibrosarcoma, given their know molecular status with *NTRK3* fusions [9]. This was the only exclusion criterion, while all age groups and ethnicities were incorporated.

All methods were carried out in accordance with relevant guidelines and regulations.

As it was a retrospective study on archival material, no informed consent of the patients could be obtained.

We received approval by the Ethics Committee of the Antwerp University Hospital/University of Antwerp (EC 18/45/517) to use historical samples without additional informed consent from the patients.

### Immunohistochemistry

As a reference method we used the VENTANA pan-TRK assay (clone EPR17341) performed according to the instructions of the vendor on a Benchmark Ultra (Ventana Medical Systems, Tucson, AZ, United States). This widely used EPR17341 clone is reactive with a conserved proprietary peptide sequence from the C-terminus of TRKA, TRKB and TRKC, and is therefore reactive with any of the oncogenic TRK proteins, although a lesser sensitivity for *NTRK3* fusions is described.

We looked at the expression of TRK in the tumor cells. All stained slides were assessed and scored independently by two pathologist (VS. and PP). Tumors were considered positive if ≥1% of tumor cells exhibit staining at any intensity above background [11, 12]. In addition, the different subcellular staining patterns (cytoplasmic, membranous, nuclear and peri-nuclear) were all considered to be positive. Moreover, for the evaluation of the immunohistochemical staining we also used the modified Histoscore (H-score) [13]. This is a semiquantitative assessment of both intensity and staining and the percentage of positive cells.

Evaluation of the slides was based on scanned slides at a Philips platform.

### Molecular Techniques

The *NTRK* fusion status and possible fusion partner of the samples was confirmed by next generation sequencing (NGS). Targeted RNA-based NGS was conducted with the Oncomine Focus Assay (OFA) panel (Thermo Fisher Scientific, San Francisco, CA, United States) on an S5 instrument, according to the manufacturer’s recommendations.

## Results

### Pan-TRK Immunohistochemical Expression in Diverse Tumor Types and Correlation With *NTRK* Fusions

We investigated the immunohistochemical expression of TRK in diverse types of soft tissue and bone tumors. Tumor characteristics are summarized in Table 1.

**TABLE 1:** Tumor characteristics.

Characteristics	Number (n)
Gender	
Male	46
Female	24
Tumor location	
Bone	19
Deep soft tissue extremities	17
Deep soft tissue trunk and back	5
Deep soft tissue head and neck	4
Skin and subcutaneous fat tissue	12
Abdomen	6
Mediastinum	5
Retroperitoneum	2
Histological type	
Chondrosarcoma	10
Ewing sarcoma	3
Osteosarcoma	5
Angiosarcoma	7
Kaposi sarcoma	8
Leiomyosarcoma	5
Liposarcoma	9
Myxofibrosarcoma	5
Rhabdomyosarcoma	3
Synovial sarcoma	3
Sarcoma NOS	12
Grade	
High grade	48
Low grade	18
Not known	4
Local or metastatic disease	
Local aggressive	15
Monometastatic disease	11
Multimetastatic disease	13
No local recurrence or metastatic disease reported	31
Oncogenic mechanism	
Oncogenic mechanism known	14
Not known oncogenic mechanism	48
Oncogenic virus (HIV)	8
Survival	
Alive	42
Death from disease	22
Death from other cause	6
Medical history	
No medical history	56
HIV	5
Lymphoma and HIV	1
Lymphoma and other tumors	1
Epithelial tumor	5
Melanoma	1
Syndrome	1
Therapy	
Excision only	28
Excision + adjuvant therapy^a^	28
Neoadjuvant CHMT	12
Not known	2

Abbreviations: n, number; NOS, not otherwise specified.

a Adjuvant therapy was either chemotherapy or radiotherapy or immunotherapy or targeted therapy or protontherapy or their combinations.

Among the different tumor types, seven tumors (10%) displayed immunohistochemical positivity for pan-TRK in the tumor cells. The remaining 63 tumors showed no pan-TRK expression. The immunohistochemical expression of these seven cases according to the H-score is illustrated in Table 2.

**TABLE 2:** Evaluation of immunohistochemical positivity for pan-TRK according to H-score.

Tumor Type	Intensity of Staining	Percentage of positive tumor cells (%)	H-score	Subcellular Staining patterns
Epithelioid angiosarcoma	3+	10	30	Cytoplasmatic
Alveolar rhabdomyosarcoma	3+	20	60	Cytoplasmatic
Osteosarcoma	3+	40	120	Cytoplasmatic
MPNST	2+	15	30	Cytoplasmatic
Alveolar rhabdomyosarcoma	3+	85	255	Cytoplasmatic
Sarcoma, NOS	1+	90	90	Nuclear
Sarcoma NOS	3+	100	300	Cytoplasmatic

Abbreviations: H-score, Histoscore; MPNST, malignant peripheral nerve seath tumor; NOS, not otherwise specified.

Among the positive tumors there were two alveolar rhabdomyosarcomas, one epithelioid angiosarcoma, one malignant peripheral nerve sheath tumor (MPNST), one osteosarcoma and two spindle cell sarcomas, not otherwise specified (NOS). All but one were reported as high grade tumors. The low-grade tumor was a spindle cell sarcoma NOS.

NGS RNA analysis was performed in all positive cases. Two out of these (nearly 28,6% among the pan-TRK positive cases and 2,86% among all tumors included in this study) showed an *NTRK* fusion, while the rest did not. The characteristics of the tumors with the fusion are summarized in Table 3.

**TABLE 3:** Summary of clinical, immunohistochemical, and molecular data.

Pt	Age	Sex	Diagnosis	Location	Fusion	IHC	
						Pattern	Intensity
1	10	M	Low grade spindle cell tumor	Skin, finger	*ETV6-NTRK3*	Nuclear	Weak
2	19	M	High grade spindle cell tumor	Deep soft tissue of the lower leg	*TFG-NTRK3*	Cytoplasmic	Strong

Abbreviations: IHC, immunohistochemistry; Pt, patient; M, male.

### A Low-Grade Spindle Cell Tumor With an *ETV6-NTRK3* Fusion

The first case concerned a 10 year-old male with a cutaneous tumor on his finger. The duration of the lesion could not be determined accurately but was estimated by the parents to be six to seven years. No previous operations or other therapies were mentioned.

**FIGURE 1:**
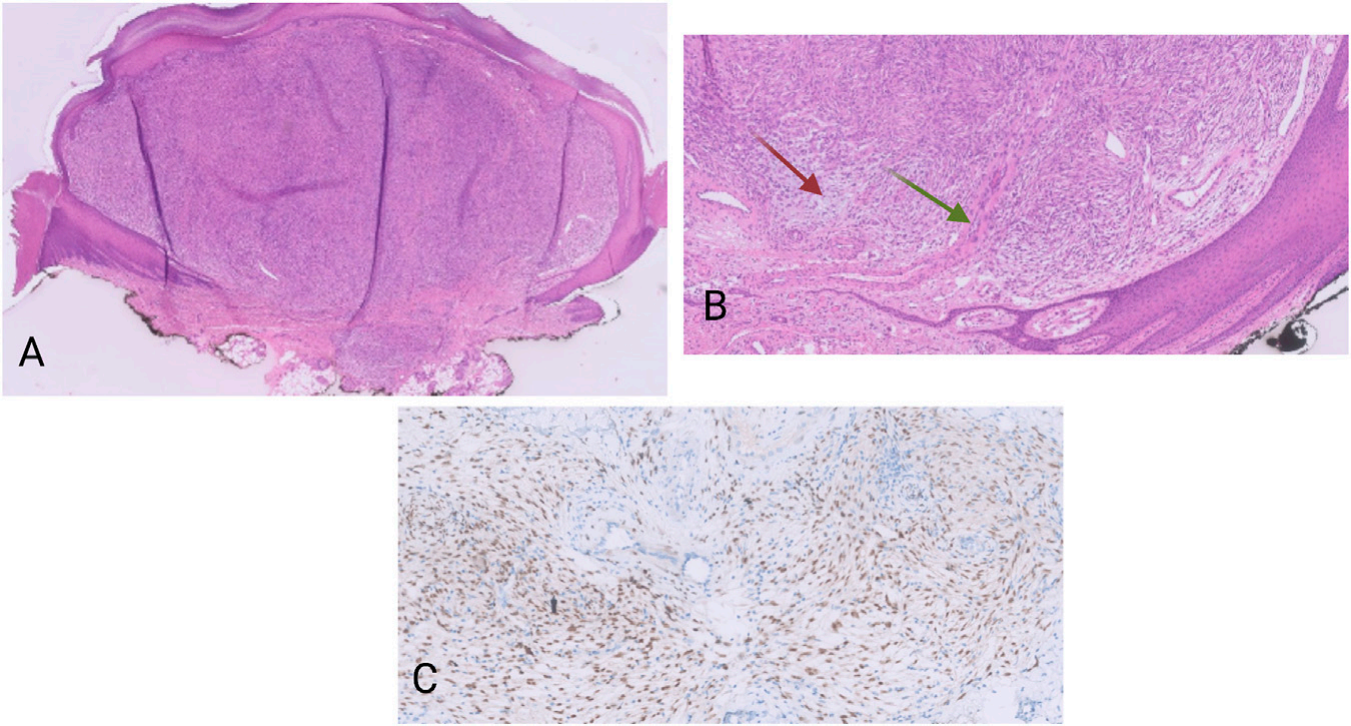
**(A)** HE, 20×. Polypoid dermal spindle cell proliferation. **(B)** HE, 100×. The tumors display a fascicular growth pattern. There are myxoid areas (red arrow), as well as vessel walls with presence of multinucleated cells (green arrow). **(C)** Pan-TRK nuclear positivity in the tumor cells. Pan-TRK assay (clone EPR17341), DAB, magnification ×200.

Macroscopically, there was a polypoid lesion that measured approximately 5 mm (Figure 1A). Microscopy revealed a dermal spindle cell proliferation. The cells were arranged in a fascicular pattern. There was some variation in size and shape but there was no striking pleiomorphism. Mitotic activity was present (up to 5 mitosis/10 high power fields), but no necrosis was perceived. There was local mucin deposition between tumor cells. Finally, some blood vessels within the tumor showed hyalinization and presence of multinucleated cells in the vessel wall (Figure 1B). A wide range of immunohistochemical stainings were performed, such as CD68, SMA, desmin, EMA, mucin 4, SOX10, S100 and CD34, from which we could not deduce a specific differentiation line of the tumor cells. More specifically, CD68 and SMA stained focally, while the rest was negative. Moreover, immunohistochemical examination for Anaplastic Lymphoma Kinase (ALK) proved to be negative.

The tumor was located in the dermis with focal extension in to the subcutis. The lesion was completely removed but with narrow margins.

Nuclear positivity of the tumor cells for pan-TRK was noticed on immunohistochemistry (Figure 1C). The NGS analysis revealed an *ETV6-NTRK3* fusion (1,328 reads out of a total of 52,464 mapped fusion panel reads). The patient underwent positron emission tomography–computed tomography (PET-CT) but no metastatic lesions could be detected. No signs of local recurrence or disseminations were noticed almost a year after the excision.

### A High-Grade Spindle Cell Tumor With a *TFG-NTRK3* Fusion

**FIGURE 2:**
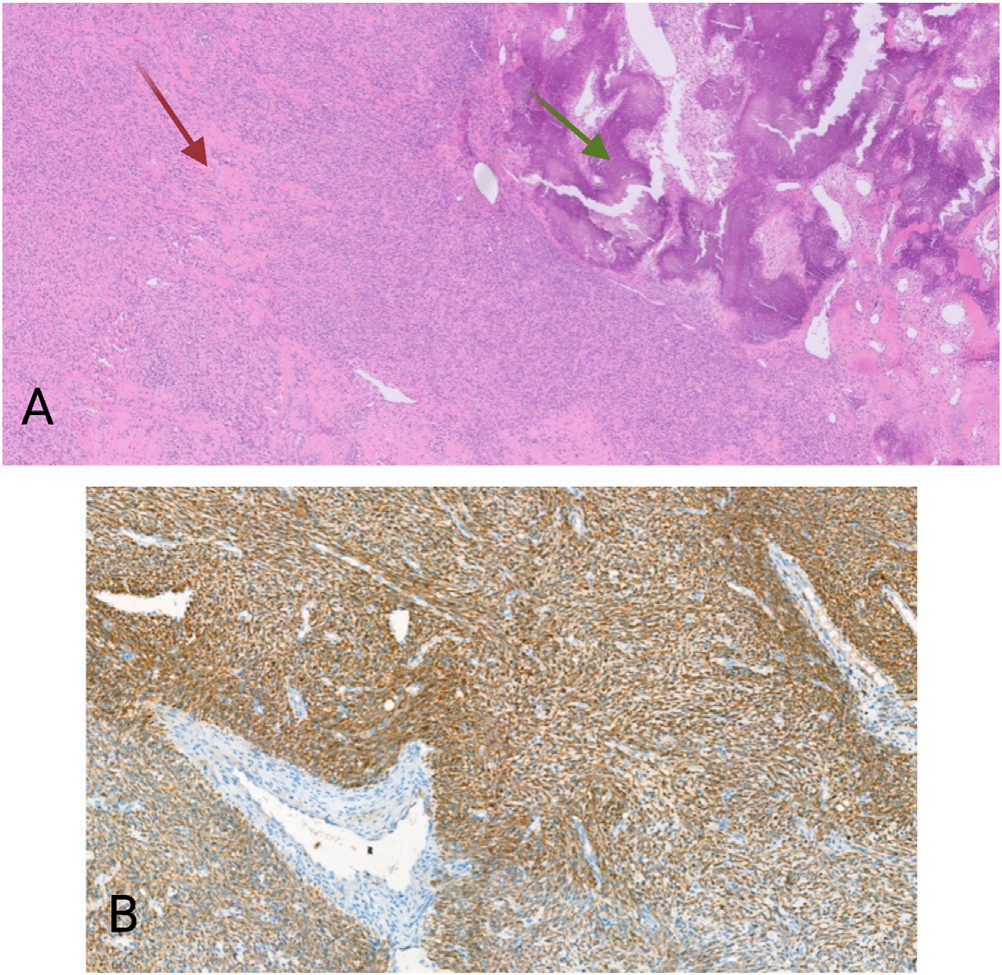
**(A)** HE, 20×. Spindle cell proliferation. Between the tumor cells are thick collagen fibers (red arrow), as well as areas with calcification and ossification (green arrows). **(B)** Pan-TRK cytoplasmic positivity in the tumor cells. Pan-TRK assay (clone EPR17341), DAB, magnification ×100.

The second case concerned a 19 years-old man. The patient presented with a tumor on the antero-external side of the right tibia dating for several months. The tumor was located intramuscularly with extension to the subcutaneous fat tissue. Three months after the first examination, the tumor was excised. Macroscopically, an almost 14 cm large mass was seen. Microscopy revealed a multinodular lesion that was partially surrounded by a thin capsule. Spindle-shaped tumor cells with a fascicular growth pattern were noticed. Thickened collagen bundles were seen in between tumor cells, as well as areas with calcification and ossification (Figure 2A). There was high mitotic activity and also necrosis. After extensive immunohistochemical analysis, including antibodies against pan-cytokeratin, S100, CD34, beta-catenin, SMA, desmin, CD10 and MDM2, the tumor was classified as high-grade spindle cell sarcoma NOS. Again here, immunohistochemistry for S100 and CD34 was negative. Despite the fact that the tumor was completely excised, given its high-grade, the patient received adjuvant radiotherapy. Four months after the excision the patient developed multiple lung metastases, which were treated with radiotherapy. Immunohistochemistry on the material from the first excision revealed a cytoplasmatic positivity for pan-TRK in the majority of the tumor cells (Figure 2B). NGS analysis showed the presence of a *TFG-NTRK3* fusion (424 reads out of a total of 2,668 mapped fusion panel reads). The patient started with larotrectinib at a dose of 100 mg twice daily. The patient was still alive a month after the initiation of treatment and was then lost from follow-up.

## Discussion

**FIGURE 3:**
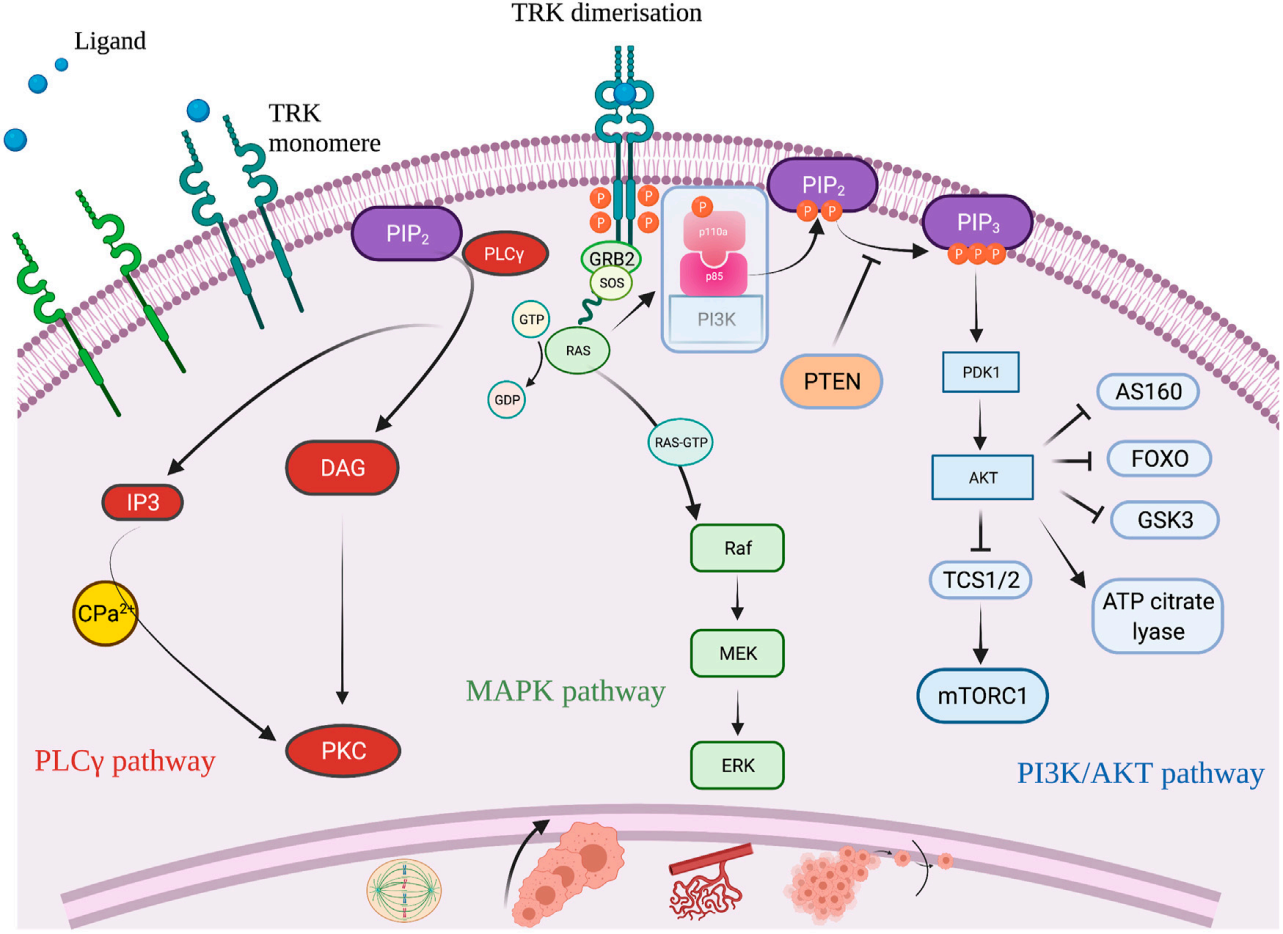
Schematic illustration of the TRK signaling pathway and its role to cell differentiation. Created with BioRender.com.

TRK is a member of the tyrosine kinase family, predominantly known for its role in neuronal cell differentiation. There are three receptors (TRK A, B, and C) encoded by the three *NTRK* genes, *NTRK 1*, *2*, and *3*, respectively [9]. TRK is a transmembrane receptor, that upon its binding with a ligand, undergoes dimerization. TRK dimerization leads to activation of three signaling pathways: the PI3K/AKT, the MAPK and the PLCγ pathway, all of which impact cell proliferation, cell growth, and cell survival [14] (Figure 3). Fusion of one of the three *NTRK* genes leads to ligand independent constitutive activation of the TRK signaling pathway that can induce uncontrolled cell growth and proliferation, neo-angiogenesis and cell migration [15].

*NTRK* fusions are highly actionable driver alterations that are found across many different tumor types. TRK inhibitors are very active against tumors that harbor one of the *NTRK* fusions. These drugs have a high response rate and durable responses [14, 15]. Hence, detection of *NTRK*-fused tumors can have important therapeutic consequences for the patient.

With regard to sarcomas, Infantile Fibrosarcoma (IF) shows the *ETV6-NTRK3* fusion in more than 90% of the cases [9]. No other specific sarcoma types are corelated with the presence of fusions of one of the *NTRK* genes. Moreover, sarcomas with an *NTRK* fusion show no other actionable alterations within the same tumor [16]. Thus, sarcomas with a known driver oncogenic alteration, as for instance alveolar rhabdomyosarcoma or synovial sarcoma, are less likely to harbor fusions of one of the three *NTRK* genes. This is in line with our results, where the two *NTRK*-fused tumors where not of a histologic type that carries a specific genetic alteration. Consequently, testing for *NTRK* fusions should be prioritized in sarcomas with high *NTRK* fusion frequency, while testing in sarcomas with a canonical oncogenic alteration might not have any diagnostic or therapeutic value [17].

Currently, correct identification of *NTRK*-fused sarcomas is only possible by means of molecular techniques. However, recently there have been attempts to describe the morphological features of tumors carrying this molecular alteration. To date, various morphological features have been described in *NTRK*-fused sarcomas, the most consistent being spindle cell morphology [10]. Growth patterns attributed to these tumors are summarized in our previous review[10] and include lipofibromatosis-like [18, 19], hemangiopericytoma-like [20], fibrosarcoma-like [21–23], neoplasms resembling inflammatory myofibroblastic tumors [24, 25] and dermatofibrosarcoma protuberans-like [26]. Moreover, spindle cell tumors with myxoid features [27] as well as characteristic blood vessels with perivascular thick collagen deposition [28] are also reported.

On a molecular basis, *NTRK* fusions are the driving oncogenic mechanisms, with fusions of the *NTRK3* gene being the most frequent, followed by fusions of the *NTRK1* gene, while only a few show *NTRK2* fusions [10].

In our case, both tumors with *NTRK* fusion presented with a spindle cell morphology. None of the tumors showed a characteristic growth pattern like those previously mentioned. It is suggested that the majority of the *NTRK*-rearranged mesenchymal neoplasms display a combination of morphological patterns, which could be a helpful clue in tumor recognition [29]. Indeed, one of the tumors that we present also showed hyalinization of the blood vessels while the other displayed thickened collagen bundles between the tumor cells.

In many cases in literature, the tumors expressed CD34 and/or S100-protein on immunohistochemistry [18–23, 26, 28, 30]. None of the *NTRK*-fused cases in our series were positive for these immunohistochemical markers. However, this could be a useful diagnostic tool in cases of a spindle cell tumor without a specific growth pattern, in order to pinpoint those neoplasms that need further investigation for the presence of the fusion.

Pan-TRK immunohistochemistry is a reliable screening method for the detection of *NTRK* fusions with sensitivity and specificity exceeding 95% [12, 31]. Careful interpretation of the immunohistochemical staining is though recommended, since its specificity is low in sarcomas with neural or myogenic differentiation, as wild-type TRK protein is physiologically expressed in neural and smooth muscle tissue [32].

In our samples, two out of the seven immunohistochemical positive cases (nearly 28,6%) correlated with the presence of a *NTRK* fusion. This is in line with a very recently published study of Nozzoli et.al., where two of the eight immunohistochemical positive cases that had available RNA material for NGS, proven to harbor an *NTRK* fusion [33]. The ratio of sarcomas with a fusion in relation to the total population of the 70 cases examined in our series is approximately 2,8%. False-negative cases, with negative pan-TRK immunohistochemistry while the tumor was proven to harbor a *NTRK* fusion, are rarely described [29, 31, 34]. The staining pattern of the antibody also varies in intensity and localization. Different subcellular staining patterns have been documented such as cytoplasmic, cell membranous, nuclear with or without peri-nuclear accentuation, all of which are considered as positive [22, 31, 35, 36]. All these staining patterns suggest the presence of the fusion, and molecular testing is needed for confirmation. Subsequently, there has been an attempt to demonstrate a correlation between the staining pattern and the *NTRK* gene fusion. Hence, it has been shown that fusions of the *NTRK1* gene mostly correlate with a diffuse cytoplasmic staining, while nuclear and rather weak pan-TRK staining is frequently mentioned with *NTRK3* gene fusions [29]. Furthermore, nuclear positivity is correlated with *ETV6-NTRK3* fusion in different tumor types [31, 37]. This is consistent with our findings, where one of the tumors showed pan-TRK weak nuclear positivity, and this tumor harbored an *ETV6-NTRK3* rearrangement.

The prognosis of *NTRK1-* and *NTRK3-* fused sarcomas in correlation with histomorphology, has also been a subject of investigation. Sarcomas with *NTRK1* gene fusions can present a low- or a high-grade histomorphology. While morphologically high-grade *NTRK1*-fused tumors display an aggressive course in the majority of the cases, morphologically low-grade *NTRK1*-fused sarcomas can have a favorable clinical course [10, 38]. On the other hand, sarcomas with fusions of the *NTRK3* gene and especially those with *ETV6* fusion partner, are mainly aggressive neoplasms, even those with intermediate cytological atypia [10, 39]. Low-grade cytomorphology is usually not a feature of these fusions. This is in contrast with our findings. We describe a tumor with *ETV6-NTRK3* fusion with a rather indolent course. The tumor was present for almost six to seven years prior to the diagnosis and showed no signs of recurrence or metastatic spread almost a year after the excision. Morphologically, the tumor showed a cellular spindle cell proliferation without pronounced cytological atypia; mitotic activity was apparent but rather limited, while no necrosis was seen. An interesting feature was the hyalinization and presence of multinucleated cells in the blood vessel wall.

In addition, we describe a *TFG-NTRK3* fused sarcoma with an aggressive course. From a histological point of view, the tumor was a high-grade neoplasm with marked cytological atypia, increased mitotic activity as well as areas of necrosis. No CD34 or S100-protein expression was observed. The patient also developed disseminated disease. These findings are again in contrast with the features of the limited *TFG-NTRK3* fused mesenchymal tumors documented in the literature [18, 39]. *TFG-NTRK3* sarcomas are amongst the rare *NTRK3-*fused sarcoma cases with a rather favorable histological and clinical picture. Opposite to our case, the tumors are known to display mostly an intermediate cytological grade or a lipofibromatosis-like morphology. Immunohistochemical positivity of the neoplastic cells with CD34 and S100-protein were documented, in contrast to our case. The prognosis of these tumors is reported favorable with no evidence of recurrence or metastasis in the (rather limited) follow up period.

Among these two tumors, the one with the best prognosis was a superficial lesion on the acral skin, while the one with the worst clinical course was located in the deep soft tissues. It therefore seems that in addition to the presence of an *NTRK* fusion, tumor location might also has a prognostic value.

### Conclusion

*NTRK* fusions are rare genetic events that can appear in a wide range of tumor types, including mesenchymal tumors. Identification of rearrangement of one of the three *NTRK* genes can lead to right treatment choices, as they show great benefit from TRK-inhibitory therapy. Nowadays, we start to recognize the histomorphology that in most cases correlates with the presence of the *NTRK* fusion in sarcomas. With our research, we aimed to broaden the diagnostic spectrum of this category and its correlation with the clinical and prognostic aspects. We described two soft tissue tumors with *NTRK3* fusions among 70 soft tissue and bone sarcomas. Both are spindle cell neoplasm from the soft tissues with variable growth patterns, in accordance with prior publications. Pan-TRK immunohistochemistry was positive, and one of the cases displayed weak nuclear staining which correlated with the *ETV6* fusion partner. In contrast to what has been described in literature, none of the tumors we present showed CD34 or S100-protein expression on immunohistochemistry. Moreover, we investigated the correlation of morphology and clinical behavior of these tumors. Namely, the neoplasm with the *ETV6-NTRK3* fusion was superficial located and displayed an indolent course, in contrast to the published cases that display an aggressive behavior. Finally, we described an exceedingly rare *TFG-NTRK3* – fused sarcoma with location in deep soft tissue that developed metastatic disease.

To conclude, *NTRK*-fused sarcomas are spindle cell tumors with variable growth patterns. Pan-TRK immunohistochemistry can pinpoint the cases that need further investigation by means of molecular testing. Nuclear positivity correlates with *ETV6-NTRK3* fusion. According to our results, the presence of specific fusion partners do not seem to be a good surrogate marker to predict prognosis. Location of the tumor (superficial versus deep) may be an additional prognostic factor.

## Data Availability

The original contributions presented in the study are included in the article/supplementary material, further inquiries can be directed to the corresponding author.
